# Draft Genome Sequence of *Desemzia* sp. Strain C1, Producing Hydrogen Peroxide, Isolated from Oil-Contaminated Soil

**DOI:** 10.1128/mra.00053-22

**Published:** 2022-05-16

**Authors:** Yongseok Ko, Youri Yang, Sunil Ghatge, Seunghyeon Kim, Hor-Gil Hur

**Affiliations:** a School of Earth Sciences and Environmental Engineering, Gwangju Institute of Science and Technology, Gwangju, Republic of Korea; University of Southern California

## Abstract

Here, we report the draft genome sequence of *Desemzia* sp. strain C1, which was isolated from oil-contaminated soil in South Korea and produces hydrogen peroxide (H_2_O_2_). The genome of *Desemzia* sp. strain C1 contains genes encoding various oxidases involved in H_2_O_2_ production and resistance to oxidative stress.

## ANNOUNCEMENT

The genus *Desemzia* includes Gram-positive, non-spore-forming, microaerophilic bacteria (1). The information on the *Desemzia* genus is limited because further novel species have not been reported since the first report by Steinhaus in 1941 (2).

**FIG 1:**
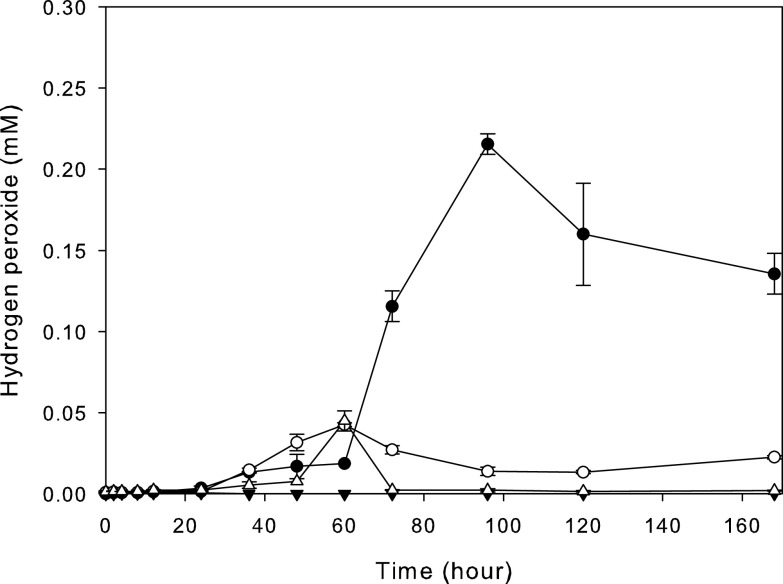
Production of hydrogen peroxide (H_2_O_2_) by *Desemzia* sp. strain C1 and Streptococcus oralis. Data for *Desemzia* sp. strain C1 cultured in BHI broth (●) and TSBY (○) and S. oralis cultured in BHI broth (▾) and TSBY (△) are shown.

Oil-contaminated soil was collected after removal with a spatula of surface soil (depth of 3 cm) from an auto repair shop in Gwangju, South Korea (35°12′22.3″N, 126°54′01.6″E). *Desemzia* sp. strain C1, producing hydrogen peroxide (H_2_O_2_), was isolated based on the Prussian blue zone-forming reaction of ferric cyanide and H_2_O_2_ produced by bacteria (3). Bacteria producing H_2_O_2_ were grown in brain heart infusion (BHI) broth (BD BBL, Sparks, MD, USA) and Trypticase soy broth containing 3 g yeast extract (TSBY) (BD Difco) at 30°C under static conditions, and the H_2_O_2_ produced was quantified using the Amplex Red hydrogen peroxide/peroxidase assay kit (Invitrogen, Waltham, MA, USA). *Desemzia* sp. strain C1 produced a maximum of 0.23 mM H_2_O_2_ in BHI broth, which was 4 times higher than that of Streptococcus oralis KACC 13048^T^, a well-known H_2_O_2_ producer, in TSBY (Fig. 1). Therefore, we sequenced the whole genome of *Desemzia* sp. strain C1 to identify genes related to H_2_O_2_ production.

Genomic DNA (gDNA) was extracted using the MagAttract high-molecular-weight (HMW) DNA kit (Qiagen, Hilden, Germany) after culture in BHI broth for 36 h at 30°C under static conditions. Extracted gDNA was sheared using the Megaruptor 3 (Diagenode SA, Liège, Belgium), and small fragments of less than 3 kb were removed using AMPure XP beads (Beckman Coulter, Pasadena, CA, USA). The DNA library was constructed by using the SMRTbell Express template preparation kit v2.0 (Pacific Biosciences [PacBio], Menlo Park, CA, USA) (4). The SMRTbell library was sequenced using the Sequel Sequencing kit v3.0 (PacBio) and a SMRT Cell 1M v2 (PacBio), resulting in 344,884 reads (*N*_50_, 8,836 bp). The draft genome of *Desemzia* sp. strain C1 was constructed based on PacBio sequencing data (5). Sequencing analysis was carried out at CJ Bioscience (Seoul, South Korea). PacBio sequencing data were assembled with SMRT Link v10.1.0.119588 according to the microbial assembly protocol (PacBio). All procedures were implemented according to the manufacturer’s protocols. Default parameters were used for all software unless otherwise specified. The resulting draft genome (average coverage, 611.0×) contained three contigs of 2,790,095 bp (*N*_50_, 2,697,877 bp), with an overall G+C content of 38.7%. The genome was annotated by the NCBI Prokaryotic Genome Annotation Pipeline (PGAP) using the best-placed reference protein set method (GeneMarkS-21) (6). Genome annotation revealed 2,582 coding DNA sequences (CDSs) and 108 RNA sequences (22 rRNA genes and 86 tRNA genes). The genome of *Desemzia* sp. strain C1 contains putative genes encoding various oxidases involved in H_2_O_2_ production, such as lactate oxidase (LOX), pyruvate oxidase, and three distinct NADH oxidases (7, 8).

The current genome information can shed light on the understanding of H_2_O_2_ production and resistance mechanisms in the bacterial system. In addition, the H_2_O_2_-producing putative gene encoding LOX could be a good candidate involved in the bacterial enzyme-mediated advanced oxidation processes to apply for degradation and detoxification of various organic pollutants.

### Data availability.

The draft genome of *Desemzia* sp. strain C1 has been deposited in GenBank under the BioProject accession number PRJNA777376, the BioSample accession number SAMN22852026, and the GenBank accession number JAJIZP000000000. The raw reads can be accessed under the SRA accession number SRR17868029. The version described in this paper is the first version, JAJIZP010000000.
